# Pleiotropy Structures Plant Height and Seed Weight Scaling in Barley despite Long History of Domestication and Breeding Selection

**DOI:** 10.34133/plantphenomics.0015

**Published:** 2023-01-30

**Authors:** Tianhua He, Tefera Tolera Angessa, Chengdao Li

**Affiliations:** ^1^ Western Crop Genetics Alliance, Agricultural Sciences, College of Science, Health, Engineering and Education, Murdoch University, Murdoch, WA, Australia; ^2^ Agriculture and Food, Department of Primary Industries and Regional Development, South Perth, WA, Australia

## Abstract

Size scaling describes the relative growth rates of different body parts of an organism following a positive correlation. Domestication and crop breeding often target the scaling traits in the opposite directions. The genetic mechanism of the size scaling influencing the pattern of size scaling remains unexplored. Here, we revisited a diverse barley (*Hordeum vulgare* L.) panel with genome-wide single-nucleotide polymorphisms (SNPs) profile and the measurement of their plant height and seed weight to explore the possible genetic mechanisms that may lead to a correlation of the two traits and the influence of domestication and breeding selection on the size scaling. Plant height and seed weight are heritable and remain positively correlated in domesticated barley regardless of growth type and habit. Genomic structural equation modeling systematically evaluated the pleiotropic effect of individual SNP on the plant height and seed weight within a trait correlation network. We discovered seventeen novel SNPs (quantitative trait locus) conferring pleiotropic effect on plant height and seed weight, involving genes with function in diverse traits related to plant growth and development. Linkage disequilibrium decay analysis revealed that a considerable proportion of genetic markers associated with either plant height or seed weight are closely linked in the chromosome. We conclude that pleiotropy and genetic linkage likely form the genetic bases of plant height and seed weight scaling in barley. Our findings contribute to understanding the heritability and genetic basis of size scaling and open a new venue for seeking the underlying mechanism of allometric scaling in plants.

## Introduction

Plant traits are often correlated, either positively or negatively, which has been ecologically explained to reflect the trade-offs in biological functions, resource allocation, and allometric scaling [1,2]. The traits could become correlated because of common evolutionary processes such as correlated selection [3]. The plant height and seed size scaling are manifested as a positive correlation between the two traits, and it sits within the broad allometric scaling that describes the relative growth rates of different body parts following a positive correlation of an individual organ versus total body size. Allometric scaling emerges as one of a seemly universal law in biology from genomes to ecosystems [4]. Over the past century, many morphological, ecological, and evolutionary size-correlated trends have been observed across organisms and life forms [5–12]. Various conceptual frameworks have been proposed when seeking the underlying mechanisms of the broadly observed allometric scaling [13–24]. The mechanism underlying the scaling law and traits correlation requires genetic explanations, which is less explored in the current research endeavors.

Pleiotropy has been proposed as an important mechanism leading to trait correlation [25]. Pleiotropy is defined as the phenomenon in which a genetic variant influences 2 or more phenotypic traits. The pleiotropic genetic variant may only have a single function, but it is involved in multiple biological processes; alternatively, the variant could have multiple functions related to different traits [25–27]. Trait correlation could also be the effect of genetic linkage, e.g., the coselection of gene variants closely linked to selected loci of interest in the chromosome leading the correlation of trait expression, as physical linkage-based correlations can be stable over many generations in species with low recombination rate [28,29]. The recent advancements of high-throughput genotyping and large-scale phenotyping offer an opportunity to decipher the different genetic basis of trait correlation. Genome-wide association studies (GWASs) reveal single-nucleotide polymorphisms (SNPs) that are significantly associated with a single phenotype [30], therefore allowing the identification of genetic variants that influence more than one trait. Furthermore, if the physical position of the responsible genetic variants on the chromosome is known, then their genetic linkage can be examined. Recently, genomic structural equation modeling (genomic SEM) emerges as a powerful method for analyzing the trait correlation through deciphering the joint genetic architecture of multiple traits. Genomic SEM assumes that all SNPs contribute to trait variations and has been shown to be more powerful than GWASs in investigating the genetic architecture of complex traits [31]. Genomic SEM models shared genetic architecture across multiple phenotypes with factors representing broad genetic liabilities through common factors analysis and systematically evaluates the pleiotropic effect of each SNP in the trait correlation network [31]. Therefore, genomic SEM offers an opportunity not only to directly explore pleiotropy as a genetic explanation of trait correlation but also to describe pleiotropic genetic variants that may have driven the trait correlation and size scaling.

Moreover, plant height and seed size (weight) are important phenotypic traits in cereal crops and pulses. The pattern and evolution of plant height and seed weight scaling might be affected by direct selection, as domestication and modern breeding often target the two traits in opposite directions. For example, shorter and stiffer stems protect cereal crops against lodging and significantly improve yield by changing the harvest index (the proportion of plant biomass in the harvested grain) [32]. Larger seeds for consumption might have been one of the selection goals in the domestication and breeding of grains and pulses [33]. What an artificial selection such as domestication and intense breeding selection affecting the pattern of size scaling remains unexplored. Barley (*Hordeum vulgare* L.) is one of the most important cereal crops in Old World agriculture and was domesticated 10,000 years ago [34]. Barley has been subjected to extensive genomic study with abundant genomic and phenotypic data resources available [35–38], which provides an unprecedented opportunity to explore the genomic basis of size scaling in plants. Here, we use the recently generated high-density genome-wide SNP profile for a diverse set of barley samples, and their measurements of plant height and seed weight to identify the possible genetic mechanisms that may lead to a correlation of plant height and seed weight to explore the genomic basis of size scaling in plants.

## Materials and Methods

### Phenotypic and genomic data and plant height–seed weight scaling

Ready-to-use phenotypic data for plant height and thousand-seed weight (hereafter seed weight) and high-density genome-wide SNP dataset for approximately 13,000 barley (*H. vulgare* L.) accessions were obtained from the Federal Ex situ Gene Bank for Agricultural and Horticultural Plant Species (IPK) in Germany. The panel includes both domesticated barley (cultivars and landraces) and its conspecific wild progenitor *H. vulgare* ssp. *spontaneum* (K. Koch) Thell. Plant height from the soil surface to the top of the spike including awns) and seed weight (in the form of thousand-seed weight) were assessed during seed regeneration using plots of at least 3 m^2^ [36]. SNP profiles were derived from a single plant of the accessions in the IPK barley collection through the genotyping-by-sequencing method [38].

We retained samples with both phenotypic and genotypic data available for further analysis. The retained phenotype and genotype data are subjected to further filtering with all samples with <10% missing genotypes and a minor allele frequency of >0.01. Consequently, we have obtained 133,588 SNPs for 12,828 samples, including wild types, landrace, and cultivars, from 85 countries and regions of all continents with agriculture. The samples also contain different habits (winter type with vernalization required for flowering or spring type with relaxed vernalization required for flowering) and growth forms (two-rowed or six-rowed) and contain sufficient variation in life history to capture the general scaling law.

Plant height and seed weight scaling was first evaluated through bivariate linear model analysis using PAST v3 [39]. If the correlation between plant height and seed weight is determined by shared genomic factors, then it would be expected that the two traits are evolutionarily correlated, independent of their phylogenetic relationship. We, therefore, first test the evolutionary correlation of the two traits after controlling phylogenetic relatedness among the samples. To do so, we first used RAxML to construct the phylogenetic tree of the 12,828 samples following a maximum likelihood procedure [40]. We then implemented a generalized least-squares regression analysis and used phylogenetic generalized analysis of variance to test the correlation of the two traits after controlling their phylogenetic relationship using the software package Phylocom [41].

### Heritability, genetic correlation, and GWASs for plant height and seed weight

We evaluated the heritability of plant height and seed weight in barley. We used a Linkage Disequilibrium and Minor Allele Frequency Stratified Genome-Based Restricted Maximum Likelihood method (GREML-LDMS) to estimate the narrow-sense SNP-based heritability (*h*^2^_SNP_) [42]. To do so, we computed linkage disequilibrium (LD) scores between SNPs with the block size of 100 kb using GCTA software [43] and then used GREML (a function within GCTA) to calculate the proportion of variance in a phenotype explained by the SNPs following an LD score regression as *h*^2^_SNP_ [42]. We further estimated the genetic correlation between the 2 traits following the bivariate GREML procedure using GCTA [43].

We further identified SNPs that are associated with either plant height or seed weight through GWAS analysis. We first calculated the first five principal eigenvectors from principal components analysis using GCTA [43] as covariates in the GWAS model to account for population genetic structure. GWAS analysis was conducted using the program FaST-LMM that calculates and uses kinship as a realized relationship matrix and following a factored spectrally transformed linear mixed model [44]. We used Bonferroni correction to determine significant SNPs.

We finally evaluated LD decay using the *r*^2^ parameter between all pairwise SNP comparisons within a genome window of 5 Mb using PLINK version 1.9 [45] and PopLDdecay [46]. The genome window of 1 Mb is estimated as the approximate distance of genetic linkage in barley [47]. We examined the pattern of the distance between immediate neighboring SNP pairs, with one SNP being significantly associated with plant height and the other with seed weight, and evaluated against the global LD decay pattern according to their distance separated in the chromosome.

### Pleiotropic effect of SNP on plant height and seed weight

GWAS summary statistic data for both traits were obtained from the above GWAS analysis and were used for common factor analysis. Pleiotropic effect of each SNP on plant height and seed weight was estimated with a common factor model [31]. The common factor model included the two traits plant height and seed weight, assuming that each SNP asserts effect on both traits**.** The effect of each SNP on each trait was estimated within a genomic SEM framework [31]. The estimate of the SNP effect and model test followed the steps as described in [48] and was implemented with GenomicSEM [31]. Experimental data are currently not compatible with the hypothesis that every mutation (or gene) affects every trait [49]. Meanwhile, large data size (e.g., >10,000 in this case) could cause spurious [50]. We identified the outliers that are deviated from the general pattern of relationship between the SNP effects on plant height and those on seed weight as pleiotropic SNPs. A bivariate linear regression between the SNP effects on plant height and those on seed weight was implemented, and residues of the regression were then obtained. The SNPs with residues of the regression beyond the 95% confident interval as outliers using a *Z* score method (*Z* score > 1.96 or *Z* score < −1.96).

Basic summary statistics were carried out using PAST v3 [39]. The R package ggplot2 [51] was used to plot model results including the Kolmogorov–Smirnov plots. Regression slopes were compared using estimated marginal means with the R package emmeans [52]. Genomic diversity (π, nucleotide diversity) was calculated using TASSEL v5 [53]. Significance was taken at *P* < 0.05 for the null hypothesis.

## Results

**Fig. 1.**
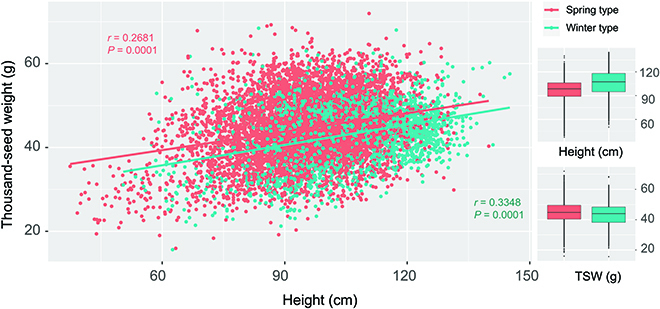
Correlation of plant height and seed weight [thousand-seed weight (TSW)].

A total of 12,828 samples have been genotyped at >90% of filtered SNP loci. There are two growth types, spring-type barley (relaxed vernalization requirement for flowering) and winter-type barley (vernalization required for flowering). Winter-type barley was taller than spring-type barley by an average of 8.8 cm, while the seed weight of winter-type barley was smaller than that of spring-type barley by a marginal 1.65 g (Fig. 1 and Table S1). For both winter-type barley and spring-type barley, plant height was correlated with seed weight with *r* = 0.268 and *P* = 0.0001 for spring-type barley and *r* = 0.335 and *P* = 0.0001 for winter-type barley, and the regression slopes did not differ (*P* = 0.680).

**Fig. 2.**
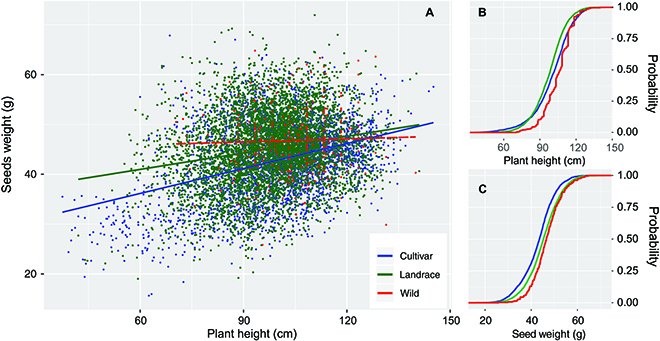
Plant height and seed weight scaling in wild and cultivated barley. (A) Pattern of scaling in wild barley, landrace, and cultivars. (B) Kolmogorov–Smirnov plot showing the phenotypic distribution of plant height in wild barley, landrace, and cultivars. (C) Kolmogorov–Smirnov plot showing the phenotypic distribution of seed weight in wild barley, landrace, and cultivars.

Both barley cultivars and landraces showed a positive plant height and seed weight scaling with *r* = 0.422 and *P* = 0.0001 for cultivars and *r* = 0.230 and *P* = 0.0001 for landraces, respectively (Fig. 2A). However, plant height and seed weight scaling was not evident in wild barley with *r* = 0.0368 and *P* = 0.5233 (Fig. 2A), which was probably due to the relatively small number of wild barley samples in the analysis. A significant difference between phenotypic distribution in wild barley, landrace, and cultivar was observed. Kolmogorov–Smi4rnov test for equal distribution for plant height between cultivar and landrace returned a *D* = 0.128 and *P* = 0.0001, and a *D* = 0.127, *P* = 0.0001 for seed weight. Domestication led to a shift to shorter status, from a median of 107.9 cm in wild barley to 98.8 cm in landraces and 102.2 cm in cultivars (Fig. 2B). Meanwhile, the shift of plant height to shorter status was accompanied by a shift to smaller seeds, as it would be expected from a positive plant height and seed weight scaling. Median seed weight decreased from 46.5 g in wild barley to 45.3 g in landraces and 43.5 g in cultivars (Fig. 2C). Meanwhile, two-rowed barley landrace, on average, had a higher seed weight than two-rowed cultivated barley, and the same was true for the six-rowed type (Table S2).

With the filtrations, a total of 133,588 SNPs were obtained for the 12,828 samples. Wild barley has a relatively higher genomic diversity (nucleotide diversity π = 0.0673, *N* = 292) than both landraces (π = 0.0535, *N* = 5,740) and cultivars (π = 0.0485, *N* = 3,390). Plant height was highly heritable with SNP-based heritability *h*^2^_SNP_ of 0.603 ± 0.095, while SNPs explained less variation in seed weight with *h*^2^_SNP_ of 0.322 ± 0.100. Plant height and seed weight were genetically correlated (*r*G = 0.272 ± 0.020, *P* < 0.0001). Barley accessions with closer genetic relatedness tended to have similar plant height and seed weight, as indicated by the phylogenetic signal lambda of 0.822 and *P* < 0.001 for plant height and the phylogenetic signal lambda of 0.853 and *P* < 0.001 for seed weight. Generalized least-squares fit by REML indicated that the two traits are phylogenetically correlated (*P* < 0.001), implying that the two traits tended to vary correlated toward a similar direction of the phenotypic spectrum.

**Fig. 3.**
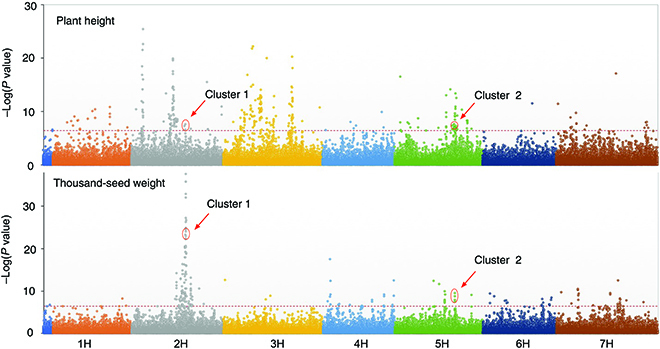
Manhattan plots of GWAS on plant height and thousand-seed weight. The two traits share eight SNPs in two clusters that are significantly associated with the traits.

GWASs revealed 314 SNPs, or 0.23% of the 133,588 SNPs in total, associated with plant height and 190 SNPs (0.14%) associated with seed weight. Among them, eight SNPs are associated with both traits (Fig. 3). The eight SNPs, forming two clusters in chromosomes 2H and 5H (Fig. 3), could be traced to at least three functional genes (Table), with two genes in cluster 1 (an expansin B3 gene and an elongation factor G gene), and one gene in cluster 2 (vacuolar protein sorting-associated protein 18 gene). Alleles of the row-type architecture genes could affect seed weight (grain size). Row type is genetically determined by the *VRS* locus [53] and Int-c (*HvTB1*) [54]. However, variations in *Vrs1* locus (*HvHox1) and HvTB1* were neither associated with seed weight nor with plant height.

**Table.** SNP loci are contributing to the positive correlation of plant height and seed weight in barley. The light green shaded loci were from joint genetic architecture analysis, the light blue shaded were from GWAS analysis. Red loci highlighted SNPs were detected in both analyses. CHR: chromosome; blank cells indicate relevant information is not available.

**CHR**	**Position**	**Gene**	**Variant description**	**Gene function description**
2	632650078			
2	651378029	HORVU2Hr1G092190	missense_variant|p.Leu574Phe	Hsp70–Hsp90 organizing protein
2	651535950	HORVU2Hr1G092260	3_prime_UTR_variant	
2	651535959	HORVU2Hr1G092260	3_prime_UTR_variant	
2	651676765			
2	651766833	HORVU2Hr1G092280	intergenic_region	
2	652633227			
2	652633251			
2	652633257	HORVU2Hr1G092360	intergenic_region	
2	653986096	HORVU2Hr1G092530	upstream_gene_variant	
2	654161868	HORVU2Hr1G092600	upstream_gene_variant	
2	654165703	HORVU2Hr1G092600	3_prime_UTR_variant	
2	654165718			
2	654165739	HORVU2Hr1G092600	3_prime_UTR_variant	
2	654786939			
2	654787214			
4	17598761			
2	647258179	HORVU2Hr1G091170	missense_variant|p.Ser1Pro	Expansin B3
2	651372029	HORVU2Hr1G092180	synonymous_variant |p.Glu123Glu	Elongation factor G
2	651766828	HORVU2Hr1G092280	intergenic_region	
2	652420092	HORVU2Hr1G092340	downstream_gene_variant	
5	593491075			
5	593533461	HORVU5Hr1G093980		Disease resistance protein
5	593534746	HORVU5Hr1G093980		Disease resistance protein
5	593561940	HORVU5Hr1G093980		Vacuolar protein sorting-associated protein 18

**Fig. 4.**
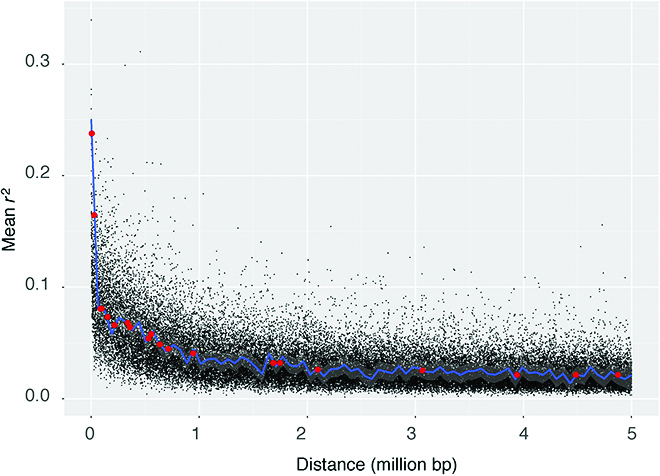
The pattern of linkage decay. Red dots highlight the SNP pairs that are significantly associated with plant height and seed weight.

LD decay analysis showed that genes in a genomic block within 1 Mb are likely linked in inheritance [47]. Of the 23 immediate neighboring SNP pairs with one SNP being associated with plant height and other with seed weight, 12 pairs were within the distance of 5 Mb in the same chromosome, and further 7 pairs were within 5 Mb of linkage block (Fig. 4), disproportional higher than expected random distribution on the chromosome (χ^2^, *P* < 0.05).

**Fig. 5.**
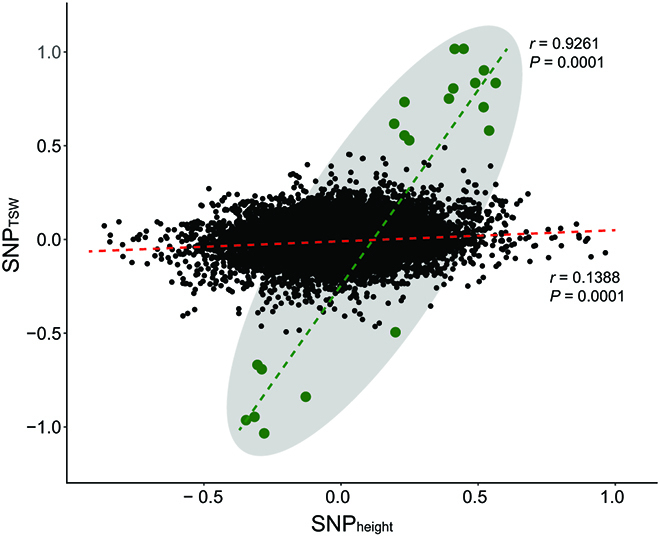
Putative effect of each SNP on plant height and thousand-seed weight. Green dots represent the identified outliers that confer significant pleiotropic effects on the 2 traits.

Common factor analysis on the pleiotropic effect of SNPs on both traits revealed a general trend of positive correlation of SNP effects on both traits (*r* = 0.138, *P* = 0.0001) (Fig. 5). Further outlier analysis revealed 20 SNPs with significant pleiotropic effects in both plant height and seed size. Their effect on each trait was significantly correlated with *r* = 0.926 and *P* = 0.0001. (Fig. 5). Noticeably, only three SNPs, among the 20 found to have pleiotropic effects through with common factor analysis, were associated with both traits as revealed with conventional GWAS analysis. The SNPs that were identified as being associated with both traits in GWAS analysis and common factor analysis, the variant description, and the gene where the SNP is located were shown in Table. Current literature links the responsible SNPs to several genes, including an heat shock protein Hsp70/Hsp90 organizing protein gene, an expansin B3 gene, an elongation factor G gene, and a vacuolar protein sorting-associated protein 18 gene (Table).

## Discussion

The offspring size at independence has been shown to be correlated with adult size across plant species [55] and animals [7,56]. Here, we show that the size scaling law holds within domesticated crop species when examining the plant height and seed weight within 12,828 globally collected barley samples. The observed correlation across species between adult size and offspring size at independence (seed weight in the case of the plant) has been hypothesized as the result of evolutionary coordination between the two traits [10,21,24], although Grubb et al. [20] argued for a biomechanically constrained mechanism shaping size scaling. Despite the fact that the mechanisms underlying size scaling remain debated from a theoretical perspective [18], the size components—plant height and seed weight—are undoubtedly under natural selection or artificially selection in the case of crop plants [58]. It is a logical hypothesis that the correlation between the two traits has a genetic basis. Using a large dataset with genome-wide SNP map and phenotypes for barley, we demonstrated that two genetic mechanisms might be involved in shaping the plant height and seed weight scaling. Both plant height and seed weight are complex traits and are influenced by multiple genes. Multiple genes with significance in plant growth and development assert pleiotropic effects on both plant height and seed weight contribute to the positive correlation between the two traits. Meanwhile, many of the genes influencing either plant height or seed weight are closely linked in the chromosome, leading to coinheritance of the two traits and contributing to the trait correlation in barley. Our results, thus, provide direct empirical evidence supporting the theoretical model, assuming that pleiotropic quantitative trait loci (QTLs) and genetic linkages affect allometric relationships [58,59]. Together, our results demonstrated that the size scaling in plant has a genetic basis, and it may be the result of shared genetic factors controlling both traits.

The plant height and seed weight scaling holds for the domesticated barley, in both landraces and cultivars, despite thousands of years of domestication and breeding targeting the two traits in opposite directions. Barley breeding tends to select varieties with shorter and stiffer for protection against lodging and the benefit of yield improvement [32]. Indeed, we observed an average shorter plant in landrace and cultivar than in wild barley. On the other hand, larger and plump barley grains are favored as plump kernels could produce more beer from a given weight of malts [60]. Larger seeds would also be a selection goal as they could contribute to yield improvement. However, we observed averagely smaller seeds in both landrace and cultivars than in the wild barley. It seems that plant height and seed weight scaling is genetically constrained and less influenced by direct artificial selection.

The seed weight may be complicated by row types in barley. As six-rowed barley bears three fertile grains per triplet instead of one as in the two-rowed form, they would produce three times more grains per spike than a two-rowed spike. However, cultivated 6-rowed barleys do not produce three-fold more grain despite the potential. Furthermore, lateral rows produce smaller and lighter grains compared with their central equivalents, making the average size of six-rowed spike grain generally smaller [60]. Therefore, six-rowed cultivars produce smaller grains than those of two-rowed cultivars, which may be associated with altered assimilate partitioning [61], other than the genetic effect underlying the size scaling.

Despite current research believes that genetic architecture of size scaling and more broadly trait correlations, in general, are polygenic [25], Gardner and Latta [62] reviewed genetic correlations among quantitative traits and found that an average of only two QTLs was shared between the two correlated traits. The traditional methods of identifying causal QTLs for a trait, such as GWAS, have limits, because those methods usually rely on linkage decay among causal and noncausal variants to detect associations and, therefore, cannot directly establish the number of causal variants [63], which consequently underestimate the pleiotropic genetic variants underlying trait correlations [25]. Indeed, using regular GWAS analysis, we identified eight SNPs in two clusters (two QTLs) that possibly have pleiotropic effects on plant height and seed weight in barley. The advanced method in deciphering the genetic architecture of trait correlation, e.g., common factor analysis within the genomic SEM framework, allows us to identify SNPs that may be pleiotropic on influencing plant height and seed weight, highlighting the power of advancement of analytical methodology.

At least three genes have been revealed to likely play an important role in structuring plant height and seed weight scaling in barley, an expansins gene, an elongation factor G gene, and an Hsp90 organizing protein gene. It is known that these genes have a function for diverse traits related to plant growth and development. In barley, transcripts of these genes could be found in both grain and shoot (https://ics.hutton.ac.uk/barleyrtd/index.html). Expansins enable the local sliding of wall polymers by reducing adhesion between adjacent wall polysaccharides and have an important role in cell wall remodeling after cytokinesis. Expansins are required in plant physiological development aspects from germination to fruiting. It is known that expansins influence seed development and seed size and increase plant height, root mass, and the number and size of leaves [64–66]. Elongation factor (EF) G protein promotes tRNA translocation on the ribosome [67]. Liu et al. [68] reported that overexpressing of the EF gene (*MaEF1A*) greatly enhanced plant height, root length, and rachis and silique length by promoting cell expansion and elongation. Hsp90 organizing protein mediates nuclear-encoded chloroplast pre-proteins binding to HSP90 before chloroplast sorting [69]. Hsp90 is extensively involved in plant growth and development and has a function for diverse traits such as hypocotyl elongation, leaf size, and seed mass [70–71]. These previous molecular biology studies suggest that the plant height and seed weight scaling may be coordinated through multifunctioning genes involved in plant growth and development.

In conclusion, our results suggest that plant height and seed weight scaling could be formed through the pleiotropic effect of many genes conferring an effect on both traits and by the genetic linkage of genes with multiple functions in plant growth and development. Plant height and seed weight scaling is genetically constrained and less influenced by direct artificial selection, which could pose a serious challenge in crop breeding when targeting correlated traits in the opposite direction. Potential solutions to this challenge point to, for example, the pleiotropic genetic variants with desirable effects on two correlated traits [56]. Although we, here, only examined plant height and seed weight scaling within a species, it could be speculated that a similar genomic basis may exist to explain the often observed allometric scaling across diverse species. The recent advances at cellular to molecular levels of organization, genomic analysis and large-scale phenotyping, and research into heritability and genetic basis of size scaling could open a new venue for a grand unifying theory on allometric scaling in plants.

## Data Availability

The SNP genotype data are publicly available at https://doi.org/10.5447/IPK/2018/9. The plant height and seed weight data can be accessed at https://doi.org/10.5447/IPK/2018/10.

## Supplementary Materials

Supplementary Materials Table S1. Overall summary of the samples in their two traits plant height and thousand seeds weight. Table S2. Summary of the samples in their two traits plant height and thousand seeds weight in relation to their domestication history.
