# Supplementary material for: Pleiotropy Structures Plant Height and Seed Weight Scaling in Barley despite Long History of Domestication and Breeding Selection
Source: Plant Phenomics. 2023 Jan 30;5:0015. doi: 10.34133/plantphenomics.0015 (PMC10076058; doi:10.34133/plantphenomics.0015)
Supplement: Supplementary Materials — Table S1. Overall summary of the samples in their two traits plant height and thousand seeds weight. Table S2. Summary of the samples in their two traits plant height and thousand seeds weight in relation to their domestication history. [file plantphenomics.0015.f1.docx]

Table S1. Summary of the samples in their two traits plant height and thousand seeds weight

|  | **Height (cm)** | | **Thousand-seed weight (g)** | | **Height (cm)** | | **Thousand-seed weight (g)** | |
| --- | --- | --- | --- | --- | --- | --- | --- | --- |
|  | Spring type | Winter type | Spring type | Winter type | 2-rowed | 6-rowed | 2-rowed | 6-rowed |
| **No. accessions** | 9558 | 2970 | 7634 | 2293 | 2487 | 5392 | 2487 | 5392 |
| **Minimum** | 37.43 | 50.44 | 15.64 | 15.95 | 49.37 | 37.43 | 28.65 | 15.64 |
| **Maximum** | 139.97 | 145.12 | 68.44 | 71.95 | 145.12 | 141.00 | 71.95 | 65.05 |
| **Mean** | 96.91 | 105.72 | 43.14 | 44.79 | 99.58 | 98.35 | 46.79 | 42.58 |
| **S.D.** | 13.28 | 15.65 | 7.33 | 7.27 | 12.82 | 15.43 | 5.51 | 7.18 |
| **Median** | 98.6 | 107.48 | 44.03 | 44.8 | 100.9 | 99.12 | 45.96 | 43.03 |

Table S2. Summary of the samples in their two traits plant height and thousand seeds weight

|  | **Cultivar** | | **Landrace** | | **Wild type** |
| --- | --- | --- | --- | --- | --- |
|  | 2-rowed | 6-rowed | 2-rowed | 6-rowed | 2-rowed |
| **No. accessions** | 1304 | 1766 | 1156 | 3558 | 287 |
| **Minimum** | 28.65 | 15.64 | 34.25 | 3.22 | 29.87 |
| **Maximum** | 67.02 | 61.44 | 71.95 | 65.05 | 63.62 |
| **Mean** | 46.03 | 40.70 | 47.69 | 43.43 | 46.80 |
| **S.D.** | 5.11 | 7.46 | 5.82 | 6.90 | 6.29 |
| **Median** | 45.24 | 41.00 | 46.84 | 43.78 | 46.52 |
